# In-Situ Growth of Au on KTaO_3_ Sub-Micron Cubes via Wet Chemical Approach for Enhanced Photodegradation of p-Nitrophenol

**DOI:** 10.3390/ma12121950

**Published:** 2019-06-17

**Authors:** Shengding Chang, Muwei Ji, Changxu Yan, Kai Zhang, Qian Deng, Jian Xu, Caizhen Zhu, Bo Li, Jin Wang

**Affiliations:** 1 Graduate School at Shenzhen, Tsinghua University, Shenzhen 518055, China; csd16@mails.tsinghua.edu.cn (S.C.); jimuwei@163.com (M.J.); ycx17@mails.tsinghua.edu.cn (C.Y.); zhangkai17@mails.tsinghua.edu.cn (K.Z.); dq17@mails.tsinghua.edu.cn (Q.D.); boli@mail.tsinghua.edu.cn (B.L.); 2 Institute of Low-dimensional Materials Genome Initiative, College of chemistry and environmental engineering, Shenzhen University, Shenzhen 518060, China; jxu@iccas.ac.cn (J.X.); makingway@163.com (C.Z.)

**Keywords:** in-situ synthesis, KTaO_3_/Au hetero-nanostructures, concentration of Au source, photo-degradation of p-nitrophenol

## Abstract

KTaO_3_/Au hetero-nanostructures were synthesized by in-situ reduction of HAuCl_4_ on the surface of hydrothermally-grown KTaO_3_ sub-micron cubes. The concentration of Au source was found to be a critical factor in controlling the hetero-nucleation of Au nanoparticles on the surface of KTaO_3_ sub-micron cubes. Loading of Au particles on KTaO_3_ nanocrystals enriched KTaO_3_ additional UV-vis absorption in the visible light region. Both KTaO_3_ and KTaO_3_/Au nanocrystals were shown to be active in the photo-degradation of p-nitrophenol, while the loading of Au on KTaO_3_ clearly improved the photo-degradation efficiency of p-nitrophenol compared to that on bare KTaO_3_ nanocrystals, probably due to the improved light absorption and charge separation.

## 1. Introduction

Potassium tantalates and their derivatives are wide-band gap semiconductors that are extensively applied in many fields, including gas phase condensation, photo-transporting, photo-detector, air-treatment, photo-conducting and photo-electronic response [1,2,3,4,5,6]. Furthermore, potassium tantalates are considered one kind of the most stable photoelectric catalysts and are widely employed in photonically-driven CO_2_ reduction [7,8], water splitting and hydrogen evolution [7,8,9,10]. While potassium tantalates exhibit excellent stability in photocatalysis, their absorption locates at Ultra-violet (UV) range and limits the employment of visible light. In order to make use of solar energy with higher efficiency, various methods are explored to modify potassium tantalates, such as cations doping [11,12] and construction of hetero-structure [13,14]. For instance, porphyrin was used as mixing dyes to sensitize potassium tantalates for photo-splitting water to H_2_ or O_2_ [8]. Design and construction of hetero-structure is a facile method to enhance the light absorption and facilitate the charge separation [9,15,16,17]. Au nanoparticles were known as an effective enhancer on photocatalysis due to the charge-separation effect and their plasmonic effects [18]. Moreover, the Au nanoparticles were reported that their plasma enhancement depends on their sizes and morphologies [19,20,21,22]. Hence, controlled growth of Au nanoparticles on KTaO_3_ nanocrystals is a possible way to obtain hetero-structure photocatalyst with high activity because KTaO_3_ was considered as a stable photocatalysis.

Recently, application of photocatalysis in waste water treatment have aroused research interest by using solar energy is also considered employing in further application. For example, p-nitrophenol, which can be produced as intermediate of dyes, medicines [23] and pesticides [24], has a strong irritating effect on human skin and can cause huge damage to liver if adsorbed by respiratory tract [25]. It is not readily degradable and thus considered to be a severe wastewater pollutant. Photodegradation is a promising approach to remove hazardous p-nitrophenol. It has been shown that TiO_2_ and ZnO can be used to photochemically degrade p-nitrophenol and its efficiency can be further enhanced by constructing heterostructures [25,26,27,28]. To our best knowledge, the photocatalytic behaviour of perovskite potassium tantalate (KTaO_3_) and its heterostructures on degradation of p-nitrophenol has not been studied. Herein, we report the in-situ growth of Au of KTaO_3_ sub-micron cubes via a facile wet-chemical approach. The prepared KTaO_3_/Au hetero-nanocrystals exhibit enhanced photodegradation performance on p-nitrophenol compared with bare KTaO_3_ nanocrystals.

## 2. Materials and Methods

### 2.1. Chemicals and Reagents

Tantalum pentoxide (Ta_2_O_5_) and gold chloride tetrahydrate (HAuCl_4_·4H_2_O, 99%) were purchased from Aladdin Reagent Co., Ltd. (Shanghai, China). Potassium hydroxide (KOH), Sodium citrate and ascorbic acid (A.A.) were purchased from Alfa Aesar Co., Ltd. (Tianjin, China). Sodium borohydride (NaBH_4_) and p-nitrophenol were purchased from Sigma-Aldrich Co., Ltd. (Shanghai, China). All of chemicals and reagents in this work are of analytical grade and used without further purification.

### 2.2. Synthesis of KTaO_3_ and KTaO_3_/Au Nanocrystals

KTaO_3_ nanocrystals were synthesized via hydrothermal method. To be specific, for KTaO_3_, 1 mmol of Ta_2_O_5_ and 0.5 mol of KOH were mixed in 30 mL of water under continuous stirring for 1 h, followed by transferring into a 50 mL Teflon-lined stainless steel autoclave (Yalunda, Beijing, China) and heating at 160 °C for 16 h. After the autoclave was naturally cooled to room temperature, the sample was purified by centrifugation at 3000 rpm for 8 min and redispersed in water for several times. The final nanocrystals were dispersed in 20 mL water.

For fabrication of KTaO_3_/Au heterostructures with different Au loadings, the obtained KTaO_3_ was dispersed into 10 mL water, and 0.5 mL (1.0 mL, or 10 μL) of HAuCl_4_ (0.48 mmol·L^−1^) and 0.1 mL ascorbic acid (0.1 mol·L^−1^) solution was separately added to the above colloid dispersion. The reaction solution was further stirred for 24 h at room temperature. The product was collected by centrifugation at 3000 rpm for 10 min to remove the self-nucleated Au nanoparticles. The raw product was further purified by centrifugation at 3000 rpm for 10 min and re-dispersed in water for several times and re-dispersed in 5 mL water. 

Au nanoparticles were synthesized via hydrothermal method, 4 mL of 1% sodium citrate aqueous solution were mixed in 30 mL of water under stirring at 70 °C for 10 min. Then, 0.4 mL of HAuCl_4_ (0.48 mmol·L^−1^) was added to above mixed solution, the solution was continuously stirred at 70 °C, the reaction was stopped until the solution turned to wine red and naturally cooled to room temperature. The raw product was collected by centrifugation at 5000 rpm for 10 min and redispersed into water.

### 2.3. Characterization

X-ray diffraction (XRD) pattern of as-prepared samples was collected with a Bruker D8 X-ray diffractometer (Bruker, Billerica, MA, USA) with Cu kα (wavelength = 1.5406 Å). The scanning electronic microscopy (SEM, Carl Zeiss AG, Oberkochen, Germany) of as-prepared samples was conducted by using a ZEISS SUPRA® 55 scanning electron microscope. The transmission electron microscopy (TEM, FEI, Hillsboro, OR, USA) images were collected on FEI T12 transmission electron microscope (working at 80 kV acceleration voltage). High resolution TEM (HRTEM) and element mapping analysis were performed on Tecnai G2 F30 transmission electron microscope (Thermo Fisher Scientific, Waltham, MA, USA). The UV-vis spectra were measured on UV-2600 (SHIMADZU, Kyoto, Japan).

### 2.4. Photodegradation Measurement

KTaO_3_ or KTaO_3_/Au nanocrystals (30.0 mg) were dispersed into 100 mL aqueous solution of 10 ppm p-nitrophenol and 2 ppm NaBH_4_ by sonication for 30 min in the dark. The resulting suspension was continuously stirred under illumination of a Xe lamp with full radiation (including violet light and visual light, the wavelength range of radiation is 320–780 nm. Perfectlight, 300 W; the light power was 300 mW and the light power density was 400 mW/cm^2^). 4 mL aliquots were sampled every 60 min and centrifuged to remove catalyst particles. The collected solution was analysed with a UV-vis spectrometer. Au nanoparticles with UV-vis spectral equivalent concentration to KTaO_3_/Au heterostructures were used photodegradation measurement. The blank experiment is carried out without any catalyst, and the dark light experiment is operated without irradiation, other operations are the same as above. The stability of KTaO_3_/Au for photodegradation was characterized by recycling the catalysts after photodegradation test for 1 h and dispersing the recycled catalysts into the flesh p-nitrophenol solution for another runs. The repeat characterizations were carried out 6 run.

## 3. Results and Discussion

**Figure 1:**
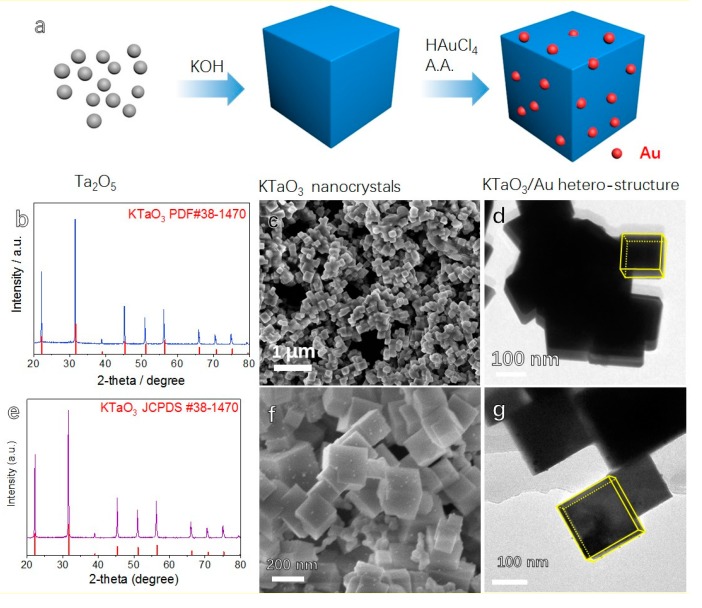
(**a**) Schematic of preparing of KTaO_3_ sub-micron cubes and KTaO_3_/Au hetero-structure; (**b**) XRD patterns; (**c**) SEM images and (**d**) TEM images of as-prepared KTaO_3_ nanocrystals; (**e**) XRD patterns; (**f**) SEM images and (**g**) TEM images of as-prepared KTaO_3_/Au heterostructure nanocrystals.

Figure 1a shows the schematic diagram of in-situ growth of Au nanoparticles on KTaO_3_ sub-micron cubes which were prepared via a hydrothermal method in a relatively high concentration of KOH solution without surfactants. As shown in Figure 1b, hydrothermally processed KTaO_3_ possess a cubic perovskite phase (JCPDS #38-1470) with good crystallinity. Figure 1c,d shows the SEM and TEM images of as-prepared KTaO_3_ nanocrystals, indicating that sub-micron cubes KTaO_3_ with an average size of ~200 nm have been obtained. Figure 1d shows the sharp edges of as-prepared KTaO_3_ nanocrystals.

By mixing the as-prepared potassium tantalate nanocrystals with an appropriate concentration of HAuCl_4_ solution and ascorbic acid solution, in-situ growth of Au on KTaO_3_ sub-micron cubes can take place. As presented in Figure 1e, the XRD pattern confirms the presence of cubic KTaO_3_ phase, while the diffraction peaks of Au are too weak to be presented due to their small size. Furthermore, the XRD pattern of KTaO_3_/Au hetero-structure confirmed that KTaO_3_ was stable during loading Au nanoparticles. The SEM image (Figure 1f) shows that the surface of cubic KTaO_3_ nanocrystals become rough because of the loading of Au nanocrystals. TEM image of (Figure 1g) cubic KTaO_3_/Au indicates that the size of loaded Au nanoparticles is about 5 nm. It is also shown in Figure 1g that Au nanoparticles grow on the surface of the KTaO_3_ nanocrystals, which agrees with the rough surface of as-prepared products after Au loading observed in SEM images. In the reported methods for synthesis of KTaO_3_ based heterostructures, surfactants were usually employed [28,29]. In our case, Au was anchored and accumulated by OH^−^ on the surface of KTaO_3_, and further grew into nanoparticles. Furthermore, the lattice mismatch between KTaO_3_ and Au is calculated (Table S1) and the largest mismatch is about 20%. The large strain on the interface between KTaO_3_ and Au prevent Au growing into a large particle and consequently, Au nanoparticles grown on the surface of KTaO_3_ will have small size.

**Figure 2:**
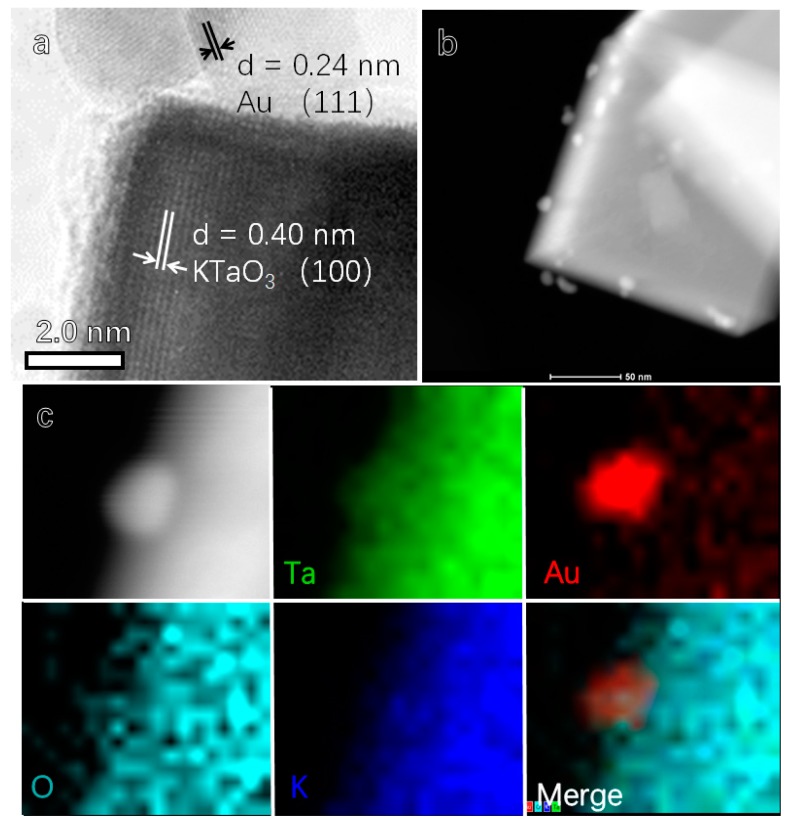
(**a**) HRTEM image, (**b**) HAADF-STEM image and (**c**) element mapping of as-prepared KTaO_3_/Au hetero-structure.

Figure 2a presents the HRTEM image of KTaO_3_/Au hetero-structure. The fringe distance of loaded nanoparticles was measured as 0.21 nm, which is consistent with the lattice spacing of (111) facets of Au. The fringe distance of the nanocube is 0.40 nm, in agreement with the lattice spacing of (100) facets of cubic KTaO_3_. The HAADF-STEM (High-Angle Annular Dark Field) images (Figure 2b) clearly confirmed the loading of Au nanoparticles on the as-prepared KTaO_3_ nanocrystals. As shown in Figure 2c, the according element mapping shows that the elements of K, Ta and O distribute in the range of the KTaO_3_ nanocube while the Au element focuses on the zone of nanoparticles, which obviously illustrates the growth of Au nanoparticle on KTaO_3_ sub-micron cubes.

**Figure 3:**
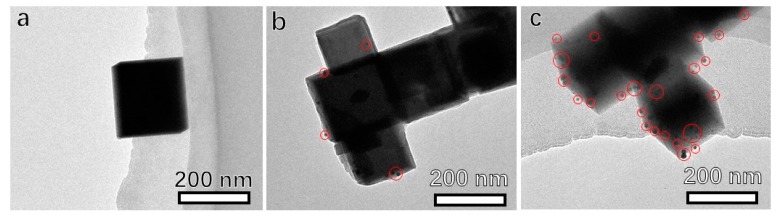
TEM images of series of as-prepared KTaO_3_/Au hetero-structure with different amounts of HAuCl_4_: (**a**) 0.005 μmol; (**b**) 0.25 μmol; (**c**) 0.5 μmol.

The concentration of Au source plays an important role in controlling the in-situ growth of Au nanoparticles on KTaO_3_ sub-micron cubes. Figure 3 shows the TEM images of series of as-prepared KTaO_3_/Au hetero-structure with different amount of HAuCl_4_ solution. By using 0.005 μmol of HAuCl_4_ (Figure 3a), almost no Au nanoparticles could be found on the KTaO_3_ sub-micron cubes, and only very few Au nanoparticles with 10 nm of size were non-uniformly formed on the KTaO_3_ nanocrystal when the employed HAuCl_4_ amount increased to 0.25 μmol (Figure 3b). As the HAuCl_4_ amount increased to 0.5 μmol, the 5 nm of Au nanocrystals were found to be well dispersed on the KTaO_3_ nanocrystal. In-situ growth of Au on the surface of KTaO_3_ sub-micron cubes is a hetero nucleation process. Insufficient amount of HAuCl_4_ would not lead to the nucleation of Au. A proper concentration of HAuCl_4_ ensured that Au^3+^ was reduced and then nucleated on the surface of KTaO_3_ nanocrystal. On the other hand, if a substantial amount of HAuCl_4_ was reduced rapidly, self-nucleation rather than hetero-nucleation on the surface of KTaO_3_ would occur.

**Figure 4:**
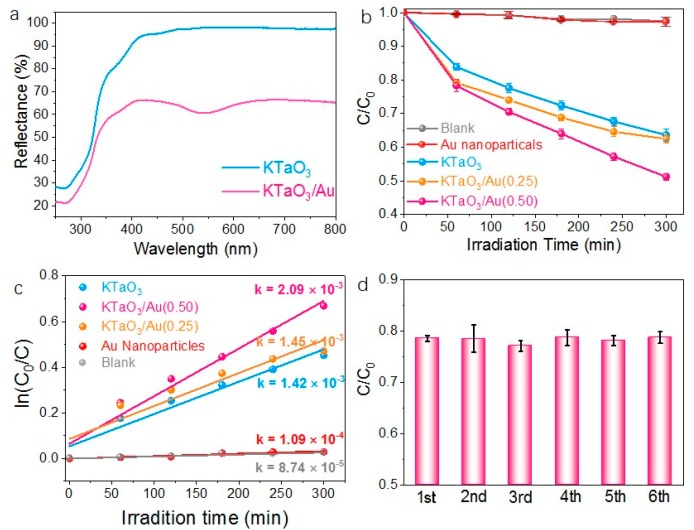
(**a**) UV-vis diffuse reflectance spectra of KTaO_3_ nanocrystals and KTaO_3_/Au hetero-structure; (**b**,**c**) photocatalytic characterization, (**d**) the photodegradation processes run by replicate.

The above results illustrate that Au nanocrystals are loaded onto the potassium tantalate nanocrystals by reducing HAuCl_4_ and well-defined hetero-structures were formed, which provide a possible way to study the Au enhancement on photocatalytic response. Figure 4a presents the diffuse reflection spectra of as-prepared potassium tantalate nanocrystals and potassium tantalates/Au hetero-nanostructures. As shown in Figure 4a, the main absorption of KTaO_3_ nanocrystals locates at ultraviolet zone (λ < 420 nm) and after Au loading, the as-prepared hetero-structures showed enhanced visible-light absorption. It is reported that the absorption range of Au nanoparticles is located at 500–550 nm, exhibiting red colour depending on their morphologies and sizes [30]. Therefore, the UV-vis spectrum of KTaO_3_/Au with enhanced absorption peak of visible light at 530 nm should result from the Au nanoparticles loading. The diffuse reflection spectra showed that after loading Au nanoparticles, the bandgap and exists-hole were changed.

As shown in Figure S1, the p-nitrophenol with KTaO_3_/Au(0.5) was placed in dark condition and the result shows that little absorption of p-nitrophenol occurred on the surface of catalysts. The photodegradation curves are presented in Figure 4b, showing that KTaO_3_/Au nanocrystals have a higher degradation efficiency than KTaO_3_ nanocrystals while little of p-nitrophenol photodegraded by using 10 nm of Au nanoparticles (Figure S2) or without using catalysts. As presented in Figure 4b, the blank case reveals that the p-nitrophenol was stable under UV-vis light irradiation and Au nanoparticle showed little capability on photodegradation of p-nitrophenol, which reveals that the capability of KTaO_3_/Au hetero-structure on photodegradation of p-nitrophenol arises from the synergetic effect of Au nanoparticles and KTaO_3_. Comparing the photodegradation of p-nitrophenol on KTaO_3_/Au with different Au loading, KTaO_3_/Au(0.5) exhibits higher activity than KTaO_3_/Au(0.25), which suggests that the more Au loading brings higher activity and confirms that the photodegradation enhancement results from Au loading. To have a better understanding of the kinetics, the photodegradation process of p-nitrophenol has been fitted to a pseudo-first-order reaction according to the following equation,

(1) $$C=C_{0}e^{-kt}$$

where *C* is the concentration of p-nitrophenol at time *t*, *C*_0_ the initial concentration of p-nitrophenol, and *k* the reaction rate constant. Just shown in Figure 4b, Au nanoparticles exhibited little activity on degradation of p-nitrophenol and after loading Au, obviously higher activity was obtained on KTaO_3_/Au hetero-structures. Furthermore, the more Au loading resulted the higher activity. Figure 4c shows the fitted curves, in which the plots between ln(*C*_0_/*C*) and *t* are presented with *k* derived from the slope of the fitted linear curve according to Equation (1). The reaction rate constants (*k*) are 2.09 × 10^−3^ min^−1^ on KTaO_3_/Au and 1.42 × 10^−3^ min^−1^ on KTaO_3_ respectively. The k value is about 20 times as the case on Au nanoparticles or blank (Figure 4c). Furthermore, the *k* of KTaO_3_/Au(0.5) is obviously higher than KTaO_3_/Au(0.25) that is a bit higher than KTaO_3_. It is thus shown the photo-degradation efficiency of p-nitrophenol on KTaO_3_ can be enhanced by forming hetero-structures with Au, due to the improved light absorption and charge separation. The photodegradation processes run by replicate of KTaO_3_/Au (Figure 4d) hetero-structure were carried out with irradiation for 60 min. The results showed that the activity of KTaO_3_/Au hetero-structure on remained well during phtodegradation, which hinted that the KTaO_3_/Au hetero-structure was stable under UV-vis irradiation. Figure 4d showed the photodegradation of p-nitrophenol run by replicate and the results showed that the activity of the KTaO_3_/Au hetero-structure remain well during photodegradation of p-nitrophenol. Just present in Figure 4d, after 6 run of photocatalysis characterization the activity of KTaO_3_/Au hetero-structure remain well, which also hinted the stability of KTaO_3_/Au hetero-structure.

## 4. Conclusions

In summary, in-situ growth of Au on KTaO_3_ sub-micron cubes has been achieved by reducing HAuCl_4_ on the surface of hydrothermally-grown potassium tantalate nanocrystals. Loading of Au nanoparticles on KTaO_3_ nanocrystals enriched KTaO_3_ additional UV-vis absorption in the red light region. Both KTaO_3_ and KTaO_3_/Au nanocrystals were shown to be active in the photo-degradation of p-nitrophenol while the loading of Au on KTaO_3_ clearly improved the photo-degradation efficiency of p-nitrophenol compared to that on bare KTaO_3_ nanocrystals. The comparison of p-nitrophenol photodegration on KTaO_3_/Au (0.5) and KTaO_3_/Au (0.25) confirmed that the Au nanoparticles loading brings enhancement on photodegration of p-nitrophenol.

## Supplementary Materials

The following are available online at https://www.mdpi.com/1996-1944/12/12/1950/s1, Figure S1: The degradation curves of p-nitrophenol with KTaO_3_/Au(0.5) in dark condition, Figure S2: The TEM image of the single Au nanoparticles, Table S1: Lattice mismatches of KTaO_3_ and Au nanoparticles.
