# Supplementary material for: In-Situ Growth of Au on KTaO3 Sub-Micron Cubes via Wet Chemical Approach for Enhanced Photodegradation of p-Nitrophenol
Source: Materials (Basel). 2019 Jun 17;12(12):1950. doi: 10.3390/ma12121950 (PMC6632096; doi:10.3390/ma12121950)
Supplement: Supplementary file 1 [file materials-12-01950-s001.pdf]

materials

MDPI

Supplementary

In-situ Growth of Au on $KTaO_3$ Sub-Micron Cubes via Wet Chemical Approach for Enhanced Photodegradation of p-Nitrophenol

Shengding Chang [1], Muwei Ji [1,2], Changxu Yan [1], Kai Zhang [1], Qian Deng [1], Jian Xu [2], Caizhen Zhu [2], Bo Li [1] and Jin Wang [1,*]

[1] Graduate School at Shenzhen, Tsinghua University, Shenzhen 518055, China; csd16@mails.tsinghua.edu.cn (S.C.); jimuwei@163.com (M.J.); ycx17@mails.tsinghua.edu.cn (C.Y.); zhangkai17@mails.tsinghua.edu.cn (K.Z.); dq17@mails.tsinghua.edu.cn (Q.D.); boli@mail.tsinghua.edu.cn (B.L.)

[2] Institute of Low-dimensional Materials Genome Initiative, College of chemistry and environmental engineering, Shenzhen University, Shenzhen 518060, China; jxu@iccas.ac.cn (J.X.); makingway@163.com (C.Z.)

* Correspondence: wang.jin@sz.tsinghua.edu.cn

Received: 30 April 2019; Accepted: 14 June 2019; Published: date

Table 1. Lattice mismatches of $KTaO_3$ and Au nanoparticles.

Au	Au (111)	Au (111)	Au (111)	Au (200)	Au (200)	Au (200)
$KTaO_3$	$KTaO_3$ (100)	$KTaO_3$ (110)	$KTaO_3$ (111)	$KTaO_3$ (100)	$KTaO_3$ (110)	$KTaO_3$ (111)
Lattice Mismatch (%)	18.1[a]	16.7[b]	2.17[b]	2.51[a]	27.7[b]	11.3[b]

[a] n= 2; [b] n = 1.

The mismatches are calculated by the definition given by the equation:

$$\text{Mishmatch} = \frac{|lattice\ parameter\ of\ KTaO_3 - n \times lattice\ parameter\ of\ Au|}{lattice\ parameter\ of\ KTaO_3}$$

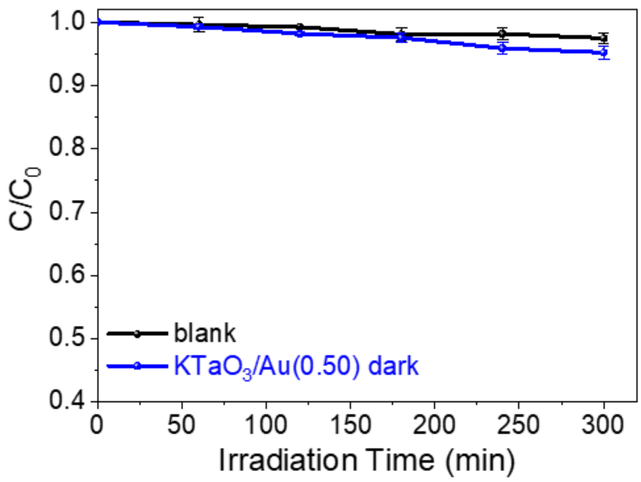

Figure 1. The degradation curves of p-nitrophenol with $KTaO_3$/Au(0.5) in dark condition.

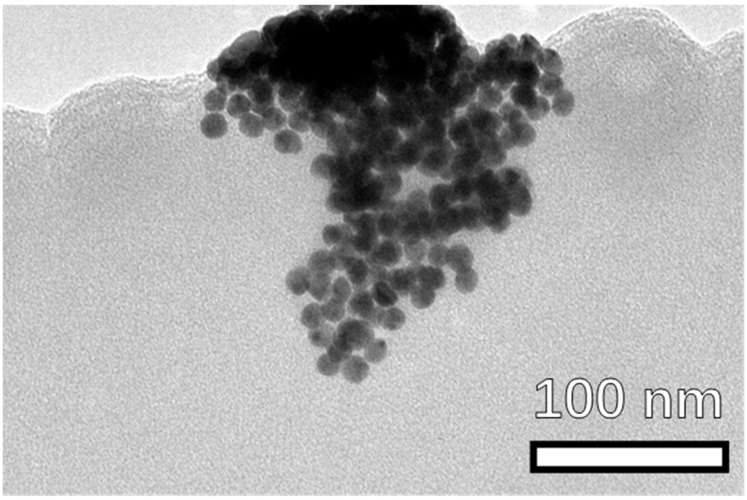

Figure 2. The TEM image of the single Au nanoparticles.

© 2019 by the authors. Submitted for possible open access publication under the terms and conditions of the Creative Commons Attribution (CC BY) license (http://creativecommons.org/licenses/by/4.0/).
